# Lucio’s Phenomenon in Hansen’s Disease: A Case Report of a Condition Not to Be Forgotten

**DOI:** 10.7759/cureus.87372

**Published:** 2025-07-06

**Authors:** Kelly A Arenas Sanchez, Pedro G Caicedo Vásquez, Lina M Sandoval Calle, José F Huertas, Jaime M Vinueza

**Affiliations:** 1 Internal Medicine, Centro Medico Nacional 20 de Noviembre, Mexico City, MEX; 2 Internal Medicine, Clínica Nueva Rafael Uribe Uribe, Cali, COL; 3 Medical School, Universidad Libre Seccional Cali, Cali, COL; 4 Medical School, Universidad Santiago de Cali, Cali, COL; 5 Clinical Research Center, Fundación Valle del Lili, Cali, COL; 6 Internal Medicine, Universidad del Valle, Cali, COL; 7 Hematology and Oncology, Universidad del Valle, Cali, COL

**Keywords:** case reports, infectious skin diseases, lepra, lepromatous leprosy, mycobacterium leprae, mycobacterium lepromatosis

## Abstract

Leprosy is a chronic infectious and granulomatous disease that occurs predominantly in tropical regions, primarily affecting the skin, mucous membranes, and peripheral nervous system. It is caused by *Mycobacterium leprae* and *Mycobacterium lepromatosis*. It is characterized by hypopigmented lesions, sensory disturbances, absence of hair in the affected area, and anhidrosis, although some of these manifestations may not be initially present. Leprosy remains a public health challenge due to its wide spectrum of clinical manifestations and leprosy-related reactions. It is a potentially curable disease, especially since the introduction of multidrug therapy (MDT) as the standard treatment, provided that diagnosis and treatment are initiated early. Lucio’s phenomenon is a rare leprosy reaction characterized by extensive necrotic skin lesions and multisystem involvement, with a high rate of morbidity and mortality. Its treatment remains challenging. Currently, the recommended therapy includes the initiation of MDT-comprising dapsone, clofazimine, and rifampicin-combined with systemic corticosteroids and specialized wound care. In cases of secondary bacterial infection, the use of antibiotics is advised based on local epidemiological data. We present the case of a patient with Lucio’s phenomenon who was treated with combined MDT and systemic corticosteroids, resulting in significant clinical improvement.

## Introduction

Leprosy, also known as Hansen’s disease, is a chronic, transmissible, infectious, and granulomatous disease classified by the World Health Organization (WHO) as one of the 20 neglected tropical diseases (NTDs) [1]. It causes significant morbidity in communities with limited access to healthcare. Leprosy is caused by *Mycobacterium leprae* (*M. leprae*) and, to a lesser extent, by *Mycobacterium lepromatosis*, which are slow-growing, strictly anaerobic intracellular microorganisms. The incubation period varies widely, ranging from several months to over 20 years, but typically falls between two and 10 years [2]. These bacteria have a predilection for Schwann cells in the peripheral nervous system and for dermal macrophages, leading to severe damage in mucous membranes, skin, and peripheral nerves. This damage results in altered sensation and mobility, which in turn predisposes patients to chronic, deep wounds and severe, irreversible structural and functional disabilities [2], as well as social exclusion due to the discrimination and stigma associated with the disease.

Leprosy is one of the oldest known diseases, with archaeological and genomic evidence dating back to 1500-3000 B.C., originating in Eastern Europe and Asia and subsequently spreading to Africa and the Americas, associated with human migration and population expansion [2]. In 2023, a total of 182,815 new cases were reported worldwide, corresponding to approximately 22.7 per 1,000,000 people. About 71.9% of these cases occurred in Southeast Asia, followed by the Americas with an incidence of 13.6%, of which 90% were reported in Brazil [3]. Factors contributing to the persistence of leprosy in endemic regions include low-income areas with limited access to healthcare, delayed diagnosis, inadequate treatment, lack of awareness, and the stigma and discrimination associated with the disease. Leprosy is primarily transmitted person-to-person via airborne nasal and oral droplets/aerosols, and less frequently through contact with exudate from eroded skin in untreated patients [4,5]. The nine-banded armadillo has been proposed as a possible wild reservoir of *M. leprae* in countries such as Mexico, Brazil, Colombia, and the southeastern United States [6,7]. Both direct and indirect exposure to armadillos is a significant risk factor for leprosy in both endemic and non-endemic regions.

Approximately 30%-50% of leprosy patients experience inflammatory reactions that complicate the natural course of the disease. These reactions may appear suddenly before, during, or after the initiation of treatment [8]. Acute lepromatous reactions can be classified into three categories: Type I reaction (reversal reaction) occurs in patients with borderline leprosy and corresponds to a type IV hypersensitivity reaction. Clinical manifestations are limited to the skin and peripheral nerves, presenting as new plaques or inflammation of pre-existing lesions, superficial ulceration, skin desquamation, facial and limb edema, burning pain, and pruritus. Type II reaction (erythema nodosum leprosum) occurs in patients with lepromatous leprosy (LL) and is a type III hypersensitivity reaction, in which immune complexes are deposited in blood vessels of the skin and internal organs, leading to leukocytoclastic vasculitis and ischemic necrosis. It is characterized by systemic involvement, presenting with fever, arthralgias, subcutaneous nodules, erythematous plaques, edema, conjunctivitis, iridocyclitis, arthritis, dactylitis, osteitis, and nephritis. Type III reaction (Lucio’s phenomenon) is observed in patients with diffuse leprosy (Lucio’s leprosy); it is a severe type III hypersensitivity reaction secondary to systemic necrotizing panvasculitis, with macrophagic infiltration of the vascular wall and direct endothelial invasion by *Mycobacterium* species. This results in painful, extensive purpuric and necrotic skin lesions, with a high risk of multiorgan failure and significant morbidity and mortality [2].

Lucio’s phenomenon was first described by Lucio and Alvarado in 1852 in Mexico and later named by Latapí and Zamora in 1948 [9]. This is a rare leprosy reaction that may occur in patients with diffuse LL, characterized by painful necrotizing skin lesions. Clinically, it manifests as erythematous or purpuric macules with irregular borders that progress to vesicles and later to superficial ulcers with angular or polygonal shapes, eventually affecting deeper tissues with necrosis. These lesions are typically associated with intense burning pain and may also present systemic symptoms such as fever, anemia, visceromegaly (hepatosplenomegaly), lymphadenopathy, epistaxis, and destruction of the nasal septum. Histopathological examination of skin biopsies reveals epidermal necrosis, aggregates of acid-fast bacilli (AFB) within endothelial cells, necrotizing vasculitis, and thrombi within blood vessel lumens [9,10].

Lucio’s phenomenon is mainly caused by *M. lepromatosis*, which plays an important role in lepromatous variants of the disease and systemic involvement [11]. Although it was once considered endemic to Mexico and Central America, numerous cases have been reported worldwide, including in China, India, Indonesia, and Taiwan [12]. The clinical picture generally presents approximately three years after disease onset [13]; in patients with clinical features compatible with leprosy and ulcerative lesions, this diagnosis should be suspected. Lucio’s phenomenon presents a clinical challenge due to its high mortality potential, complications from secondary bacterial infections, and risk of multiorgan failure. A lack of awareness about leprosy and its complications may delay diagnosis and timely treatment, increasing the risk of mortality, sequelae, and permanent deformities. Treatment includes supportive care, specific multidrug therapy (MDT) for leprosy, and in some cases, the use of corticosteroids [14-16]. In combination with prednisolone, other immunosuppressive agents such as azathioprine, cyclosporine, methotrexate, and biologic therapies (infliximab, etanercept) have shown benefits in managing type 1 and type 2 leprosy reactions. Plasmapheresis has also been reported to aid in the removal of immune complexes and is particularly recommended in patients who do not respond to conventional therapies. Thalidomide has been proposed as a therapeutic option in severe cases of Lucio’s phenomenon due to its anti-TNF-α effect; however, its use has not yet been approved. In severe and treatment-refractory cases, plasmapheresis has been considered as a therapeutic alternative due to the presence of circulating immune complexes, although further studies are required to validate this strategy [2,15].

## Case presentation

A 90-year-old male patient, residing in an urban area of Cali, Colombia, with no known medical history and retired from his occupation, presented with a seven-day history of cough, nasal congestion, and unquantified febrile episodes. Over the past five days, he developed lower limb edema and a disseminated dermatosis affecting all four extremities, characterized by multiple serous-filled blisters that spontaneously drained and progressed to erythematous-violaceous ulcers, some with necrotic areas. The patient's relative reported topical treatment with magnesium sulfate compresses without improvement.

**Figure 1:**
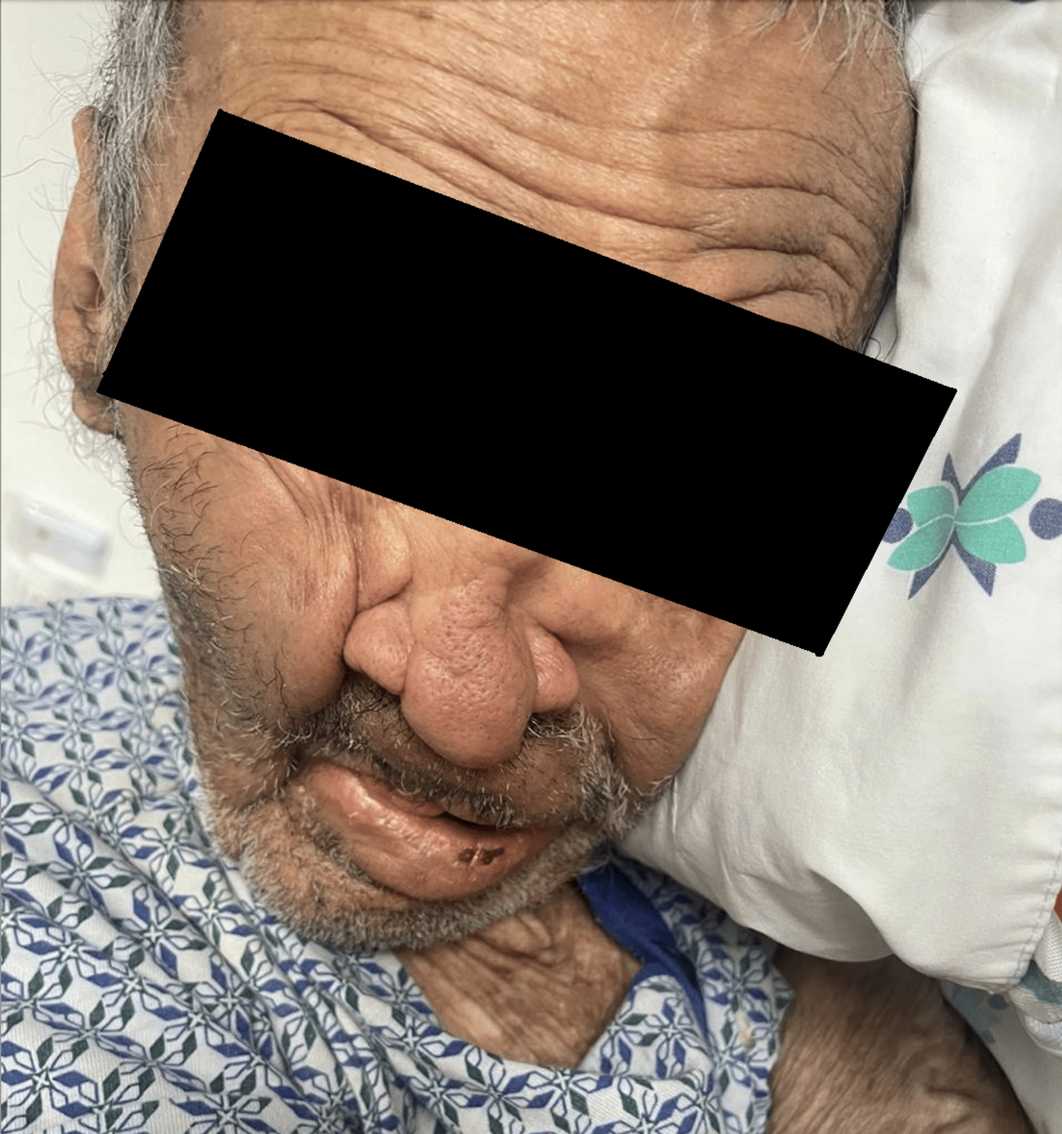
Facial appearance of the patient with leonine facies characterized by the loss of the eyebrow tails, nasal deformity with loss of the nasal septum, and mild bilateral auricular cartilage loss

**Figure 2:**
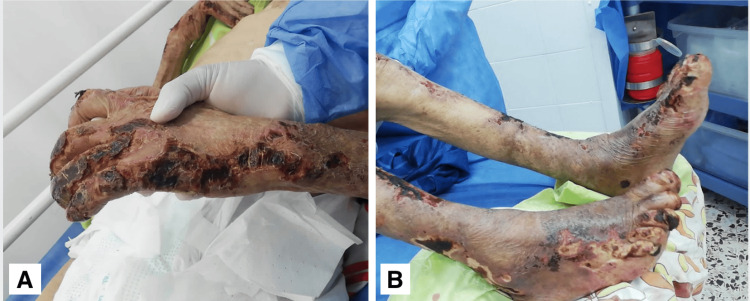
Erythematous-violaceous ulcers with irregular and asymmetric borders on the extremities (A) Upper extremity showing skin lesions characteristic of Lucio's phenomenon. (B) Lower extremities showing severe skin lesions associated with Lucio's phenomenon

**Figure 3:**
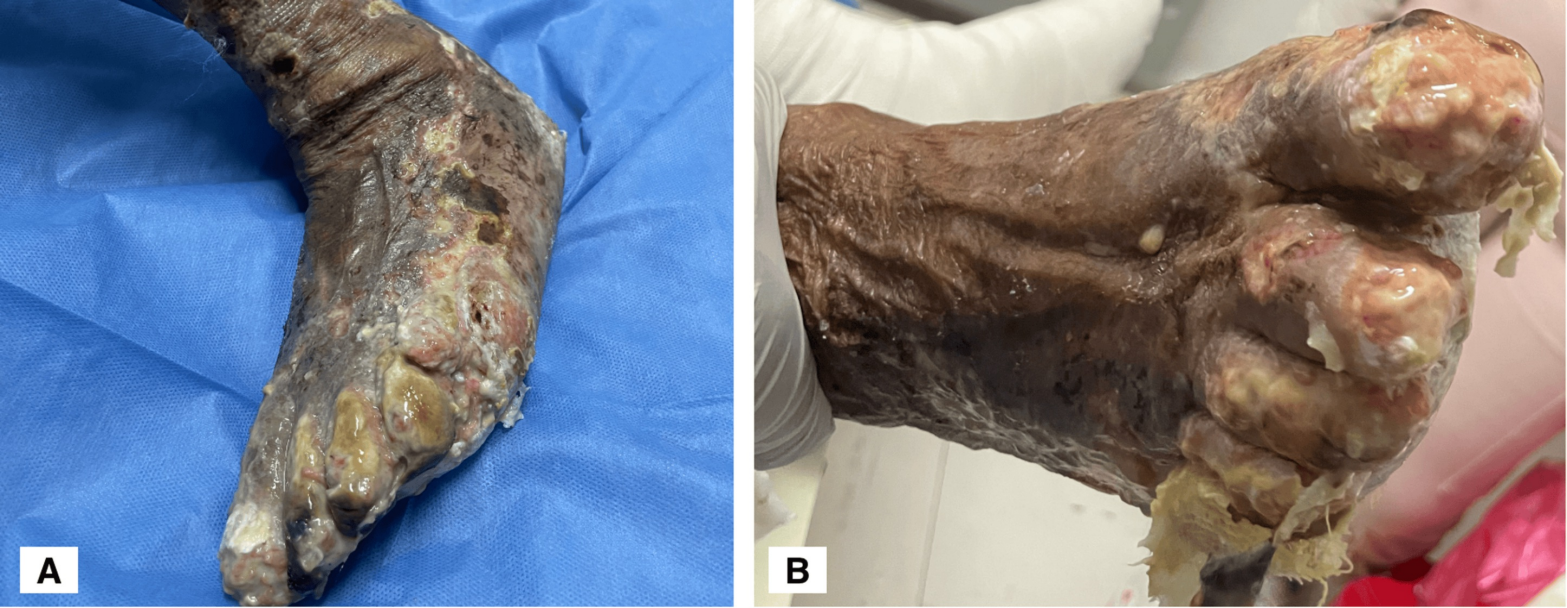
Asymmetric ulcers with irregular borders on the lower extremities, with complete absence of toenails (A) Left foot: asymmetric necrotic-based ulcers with irregular borders distributed over the left leg, dorsum, and sole of the foot. (B) Right foot: ulcers on an erythematous base with skin detachment at the level of the toe phalanges and the sole of the foot

Physical examination revealed leonine facies, loss of the eyebrow tail, nasal septum destruction, slight bilateral auricular cartilage loss, palpebral ptosis, and bilateral blindness (see Figure 1). Multiple dermatoses were identified: (1) hypopigmented macules on the anterior and posterior thorax; (2) hyperpigmented macules in the lumbosacral region; (3) lesions on the upper extremities, affecting the forearms, elbows, dorsum of the hands, the third, fourth, and fifth digits of the left hand, and the fifth digit of the right hand (Figure 2); and (4) lesions on the lower extremities, affecting the knees, anterior and lateral aspects of the legs, dorsum and soles of the feet, and toes bilaterally. The latter consisted of multiple erythematous-violaceous ulcers, some necrotic, on an erythematous background, extensive, asymmetric, with irregular, linear, and some stellate borders, draining foul-smelling serous exudate (Figure 3). No palpable nerve trunks were detected, but the patient reported intense pain on palpation of the extremities. The remaining skin, adnexa, and mucosae showed no abnormalities.

Upon admission, routine laboratory tests were performed, revealing a complete blood count without leukocytosis, neutrophilia, or lymphopenia; grade I anemia according to WHO criteria; preserved renal function; normal liver function tests; elevated lactate dehydrogenase (LDH); elevated C-reactive protein (CRP) and ESR; negative viral panel for hepatitis and HIV; and negative serology for syphilis. Leprosy was suspected, and a slit-skin smear was requested for AFB.

**Figure 4:**
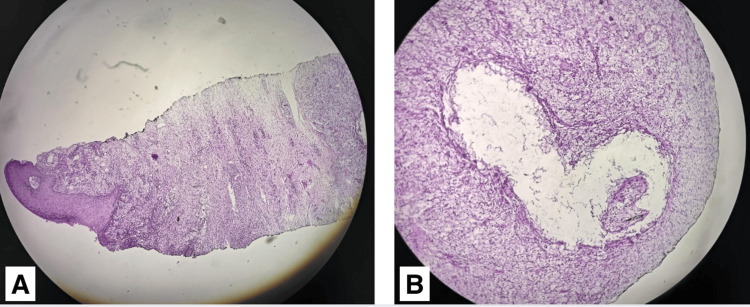
Histopathological image of the skin biopsy (A) A cross-sectional view of the epidermis and dermis is observed, showing extensive granulomatous infiltration extending into the deep dermis. Well-defined granulomas are seen, composed of epithelioid cells surrounded by a dense infiltrate of lymphocytes, with the involvement of nerve fibers. Stain: hematoxylin and eosin. (B) Granulomatous inflammatory infiltrate is visualized. Stain: hematoxylin and eosin

Following the slit-skin smear for AFB, a bacillary index of 2+ was found in both earlobes. A skin biopsy from the right leg showed epithelioid granulomas surrounded by lymphocytes affecting nerve filaments and extending into the papillary dermis (Figure 4). These findings confirmed the diagnosis of multibacillary leprosy. Based on clinical manifestations and histopathological findings, a diagnosis of Lucio’s phenomenon was also established.

**Figure 5:**
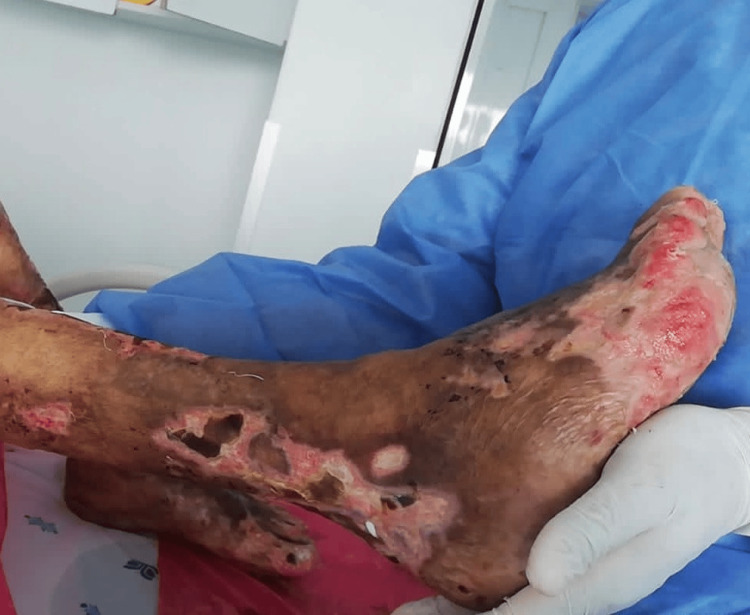
Lower extremities of the patient in recovery A favorable evolution is shown after the initiation of treatment, with reduction of necrosis, appearance of granulation tissue, and decreased purulent exudate in the affected areas

Treatment was initiated with MDT (rifampicin, clofazimine, and dapsone), systemic corticosteroids (prednisolone 1 mg/kg/day), and intravenous antibiotics (meropenem and vancomycin) for seven days. No bacterial cultures were performed prior to the initiation of antibiotic therapy. The patient showed progressive resolution of the systemic inflammatory response, with no further febrile episodes, granulation tissue formation in the cutaneous lesions (Figure 5), and improvement in pain as measured by the Visual Analog Scale (VAS) following the initiation of combined therapy. He was discharged after 13 days of hospitalization for strict outpatient follow-up by a multidisciplinary team and under the epidemiological health authority in the context of his leprosy diagnosis, aiming to complete at least 12 months of treatment.

## Discussion

Leprosy, also known as Hansen’s disease, is a chronic, transmissible, infectious, and granulomatous disease classified by the WHO as one of the 20 NTDs [1]. It is caused by *M. leprae* and, to a lesser extent, by *M. lepromatosis*-slow-growing intracellular organisms that require a prolonged incubation period (usually between two and 10 years) [2] before the onset of clinical manifestations. It primarily affects the mucous membranes, skin, and peripheral nervous system due to its tropism for dermal macrophages and Schwann cells [2]. Historically of global distribution, leprosy is currently confined to tropical regions, particularly in Southeast Asia, Central and South America, Africa, the Eastern Pacific, and the Western Mediterranean [4]. Its clinical presentation depends on the host’s immune response to infection and can be classified based on clinical and/or histopathological features into LL, borderline lepromatous (BL), tuberculoid leprosy (TT), borderline tuberculoid (BT), and indeterminate leprosy [4,5].

Approximately 30%-50% of patients with leprosy experience acute or subacute immune reactions during the natural course of the disease, during treatment, or even after its completion [8]. These leprosy reactions are clinically and histopathologically classified into type I reaction (reversal reaction), type II reaction (erythema nodosum leprosum), and type III reaction (Lucio’s phenomenon). These immune-mediated reactions significantly contribute to disability, decreased quality of life, and increased morbidity and mortality [16,17].

Lucio’s phenomenon is a type III leprosy reaction occurring within the anergic pole of the leprosy spectrum, particularly in cases associated with diffuse LL and nodular LL. It is characterized by a rapid and severe clinical course with high mortality rates [17]. It represents a type III hypersensitivity reaction, triggered by a high antigenic load that precipitates the formation of immune complexes within small- and medium-sized blood vessels. This leads to intravascular thrombosis and subsequent vasculonecrotic reactions, favoring the development of extensive cutaneous ulcerations, ischemia, infarction, and tissue necrosis [17]. It is a rare, aggressive, and diagnostically challenging presentation, especially in non-endemic areas where diagnosis is often delayed due to the wide differential diagnosis, leading to the progression of disease and associated complications.

The clinical presentation in this patient was consistent with a type III leprosy reaction (Lucio’s phenomenon), confirmed by clinical, bacteriological, and histopathological findings: cutaneous ulceration, positive slit-skin smear for AFB with a bacteriological index of 2+, and skin biopsy revealing extensive granulomatous infiltration extending into the deep dermis, with well-formed granulomas composed of epithelioid cells surrounded by a dense lymphocytic infiltrate, along with involvement of peripheral nerve twigs. A diagnosis of multibacillary LL was established, and treatment was initiated with MDT and systemic corticosteroids. Broad-spectrum antibiotics were administered due to the risk of secondary bacterial infection, although cultures were not obtained prior to antibiotic initiation.

Prognosis and mortality are difficult to predict due to the limited number of reported cases in the literature and the high mortality rate, particularly associated with multiorgan failure. Currently, there is no standardized treatment protocol for Lucio’s phenomenon, and published data on its management are scarce, likely due to its rarity and the associated high morbidity and mortality. Recommended treatment includes MDT (rifampicin, clofazimine, and dapsone) as per WHO guidelines: rifampicin (600 mg monthly), clofazimine (300 mg monthly), dapsone (100 mg daily), and clofazimine (50 mg daily) for one year [1]; systemic corticosteroids such as intravenous prednisone at 0.5 mg/kg/day; and specialized wound care [14-16]. Continued medical education and training regarding the clinical spectrum of leprosy remain crucial for early diagnosis and timely initiation of treatment. As part of its global zero-leprosy strategy, WHO also emphasizes the importance of community education regarding diagnosis and treatment adherence [1].

## Conclusions

Leprosy remains an underdiagnosed, neglected, and non-eradicated disease. Therefore, strengthening public health education at the global level is essential to achieve early diagnosis and timely treatment. It is a treatable and curable condition, and early diagnosis significantly reduces sequelae and deformities. Enhanced awareness of Lucio’s phenomenon among general practitioners and dermatologists is essential to facilitate earlier diagnosis, mitigate complications, and improve patient outcomes. Close follow-up of patients undergoing treatment is crucial, given the high incidence of leprosy reactions and associated complications.

Lucio's phenomenon is a rare lepromatous reaction with a potentially fatal clinical course. It requires early and timely intervention to reduce both mortality and irreversible sequelae and deformities. Currently, no specific medical treatment has been established for this condition. However, MDT (rifampicin, clofazimine, and dapsone), in combination with systemic corticosteroids and specialized wound care, may be beneficial. Despite this, its progression can be severe and unpredictable, highlighting the need for further research.
